# Laryngeal Transplantation in Cancer Patients: Evaluation of Surgical Outcomes and Functional Recovery

**DOI:** 10.1002/mco2.70748

**Published:** 2026-04-26

**Authors:** Zheng Jiang, Mailudan Ainiwaer, Pengwei Zhao, Yansheng Hu, Xin Yang, Yu Xiong, Bin Zeng, Longhao Wang, Jun Liu, Fei Chen

**Affiliations:** ^1^ Department of Otolaryngology‐Head & Neck Surgery West China Hospital Sichuan University Chengdu China; ^2^ Head and Neck Surgical Center West China Hospital Sichuan University Chengdu China; ^3^ Department of Biotherapy West China Hospital Sichuan University Chengdu China; ^4^ West China Lecheng Hospital Sichuan University Qionghai China

**Keywords:** hypopharyngeal cancer, immunosuppression, laryngeal cancer, laryngeal function, laryngeal transplantation

## Abstract

Laryngeal transplantation offers a promising avenue for functional restoration following total laryngectomy, yet concerns regarding cancer recurrence and perioperative challenges persist. This pilot study evaluates the preliminary outcomes of a refined surgical technique and postoperative follow‐up protocol specifically for cancer patients. Between 2023 and 2024, four patients underwent laryngeal transplantation at West China Hospital, with immunosuppressive therapy, microbial prophylaxis, and postoperative rehabilitation. Over a follow‐up period ranging from 10 to 26 months, outcomes were heterogeneous. Two recipients (T4 laryngeal squamous cell carcinoma) achieved excellent functional recovery in swallowing and phonation with sustained rejection‐free survival for 20 and 23 months and disease‐free survival for 20 and 26 months, respectively, though both remained tracheostoma dependent. One of these survivors is currently undergoing treatment for graft rejection after unilateral cessation of immunosuppressants. The remaining two patients succumbed to severe pneumonia with sepsis and tumor recurrence at 10 and 11 months posttransplant. Functional assessments in surviving cases indicated progressive nerve regeneration, with optimal voice and swallowing outcomes achieved approximately 6–8 months postsurgery. Ultimately, these findings demonstrate the technical feasibility of laryngeal transplantation in a pilot cohort of cancer patients, providing critical data for future protocol refinement.

## Graphical abstract

Our pilot clinical study in laryngeal transplantation achieved divergent outcomes: two cases maintained functional speech/swallowing with around 2 year graft survival and have been ongoing, while two succumbed to sepsis (10mo) and hemorrhagic recurrence (11mo). Refined microsurgical techniques and dynamic immunosuppression enabled functional recovery by 6–8 months in survivors, demonstrating procedural feasibility while highlighting persistent oncologic and immunosuppressive challenges requiring protocol optimization.

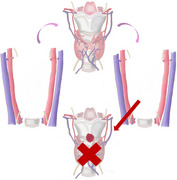

## 1 Introduction

Laryngeal and hypopharyngeal squamous cell carcinoma (SCC) are prevalent head and neck malignancies worldwide, for which total laryngectomy (TL) remains a curative mainstay for locally advanced, recurrent, or functionally inoperable disease. Despite reliable oncologic control, TL causes irreversible loss of three core laryngeal functions—phonation, deglutitive protection, and spontaneous respiration—with profound, lifelong impacts on health‐related quality of life, psychological well‐being, and social integration [1]. This functional devastation creates a critical unmet clinical need in post‐TL rehabilitation for head and neck cancer survivors.

Current rehabilitative strategies for post‐TL patients, including tracheoesophageal puncture, esophageal speech, and electrolarynx, only partially restore verbal communication, with inherent limitations that prevent replication of the native larynx's integrated physiologic functions. Vascularized composite allotransplantation of the larynx, by contrast, is the only therapeutic modality capable of restoring near‐native laryngeal structure and function, offering a transformative alternative to conventional rehabilitation [2]. Since the landmark long‐term successful laryngeal transplant reported by Strome et al. in 1998 [3, 4], subsequent cases from centers in the United States, Poland, and most recently the French ECLAT team and Mayo Clinic in 2023–2024, have consistently validated the technical feasibility and reproducibility of this procedure [5, 6, 7, 8]. However, widespread clinical translation remains hindered by unresolved challenges, most notably its application to the oncologic population.

Oncologic patients represent over 90% of individuals undergoing TL globally, yet laryngeal transplantation in this cohort remains ethically and clinically controversial. Unlike candidates with traumatic laryngeal loss or benign laryngotracheal stenosis, cancer survivors require lifelong immunosuppression, which carries a theoretical risk of impairing tumor immunosurveillance, accelerating recurrence, or unmasking occult micrometastases [9, 10]. This creates a core dilemma: a nonlife‐saving, quality‐of‐life‐focused procedure with potential oncologic risk. Despite these concerns, seminal work by McNeil et al. demonstrated that most laryngeal cancer patients are willing to trade measurable life expectancy for restoration of functional laryngeal speech [11], and real‐world data from lung transplantation in thoracic malignancy patients show comparable survival between carefully selected transplant recipients and conservatively managed patients [12], challenging the assumption that immunosuppression is inherently incompatible with oncologic safety. Even so, robust clinical data on laryngeal transplantation in the oncologic population remain extremely scarce.

Existing literature largely focuses on technical feasibility in nononcologic cohorts, with limited detailed reporting of standardized perioperative management, immunosuppression and rejection monitoring protocols, and long‐term oncologic and functional outcomes in cancer patients. Our group has previously published preliminary data on the first successful laryngeal allotransplantation in China, as well as an analysis of thyroid graft viability in the four‐patient cohort described herein [13, 14]. Building on this foundational work, this study presents our refined surgical technique, standardized perioperative management protocol, and comprehensive evaluation of surgical, oncologic, and functional outcomes in four patients with laryngeal or hypopharyngeal SCC undergoing laryngeal transplantation. We aim to provide critical preliminary proof‐of‐concept data, address persistent clinical and technical pitfalls, and establish a replicable framework for laryngeal transplantation in appropriately selected oncologic patients.

## 2 Results

### 2.1 Demographics and Perioperative Characteristics

Four male cancer patients (mean age 56.0 ± 5.24 years) underwent laryngeal transplantation between April 2023 and May 2024 (Table 1). Three patients had advanced laryngeal SCC, while one had hypopharyngeal SCC. One patient had a prior history of open partial vertical laryngectomy for SCC, and another had undergone laryngoscopic laser surgery. All patients underwent tracheostomy prior to transplantation. Neoadjuvant chemotherapy, including immune checkpoint inhibitors in two cases, was administered to reduce tumor burden. The mean operating time was 588.5 ± 91.69 min.

**TABLE 1:** Basic demographics and clinical conditions of four patients.

Patient no.	Age	Sex	Cancer type and staging	Baseline chronic conditions	Prior surgeries	Neoadjuvant therapy
1	65	M	Laryngeal SCC (rT3N0M0)	/	Right vertical laryngectomy; Emergency tracheostomy	/
2	52	M	Hypopharyngeal SCC (T3N3M0)	Hypertension, diabetes, coronary heart disease	Emergency tracheostomy	2 circles of albumin‐bound paclitaxel + cisplatin + tislelizumab
3	53	M	Laryngeal SCC (T4aN0M0)	Fatty liver, pustular psoriasis	Emergency tracheostomy	2 circles of albumin‐bound paclitaxel + cisplatin
4	54	M	Laryngeal SCC (rT4aN0M0)	Fatty liver	Rigid laryngoscopic laser surgery; Emergency tracheostomy	2 circles of albumin‐bound paclitaxel + cisplatin + cadonilimab

### 2.2 Survival and Oncologic Outcomes

The primary outcomes were presented in the Table 2.

**TABLE 2:** Clinical outcomes of four patients.

Patient No.	Intra& postoperative complications	Follow‐up period	Disease‐free survival	Rejection‐free survival
1	1. Mild hemorrhage around the tracheostoma 2. Bacteremia 3. Pharyngocutaneous fistula 4. Posttransplantation diabetes mellitus 5. Lower limbs thrombosis 6. Drug‐related acute kidney injury 7. Hypoalbuminemia, anemia	10 months	10 months	10 months
2	1. Hyperthyroidism 2. Irritable bowel syndrome (alternative constipation and diarrhea) 3. Transient delirium 4. Wound infection 5. Hypoalbuminemia, anemia 6. Perihepatic effusion 7. Right pleural effusion	11 months	7 months	11 months
3	1. Anemia 2. Hypoalbuminemia	20 months	20 months	20 months
4	1. Pneumonia 2. Nausea/vomiting 3. Transient delirium 4. Anemia, hypoalbuminemia 5. Allergic dermatitis	26 months	26 months	23 months

Postoperative follow‐up demonstrated heterogeneity in survival and complication rates. The 65‐year‐old recipient (rT3N0M0 laryngeal SCC) succumbed to multiple organ failure secondary to severe systemic infection 10 months posttransplant. Complications in this patient began at the 7th postoperative month with a right epididymal hematoma and testicular abscess (*Klebsiella pneumoniae*), requiring orchiectomy. Subsequent complications included severe lung infection, Gram‐negative bacillemia, and coinfections with *Pneumocystis* and *Candida*.

The 52‐year‐old recipient (T3N3M0 hypopharyngeal SCC) developed a postoperative thyroid storm controlled with propylthiouracil. This patient maintained stability on a triple immunosuppressive regimen. However, tumor recurrence occurred at 7 months, characterized by a neck mass and edema. Despite tumor regression following combined radiotherapy and immunotherapy (ivonescimab 500 mg + cetuximab 600 mg), the patient died 11 months postoperatively due to fatal hemorrhage at the tracheostomy site.

**FIGURE 1:**
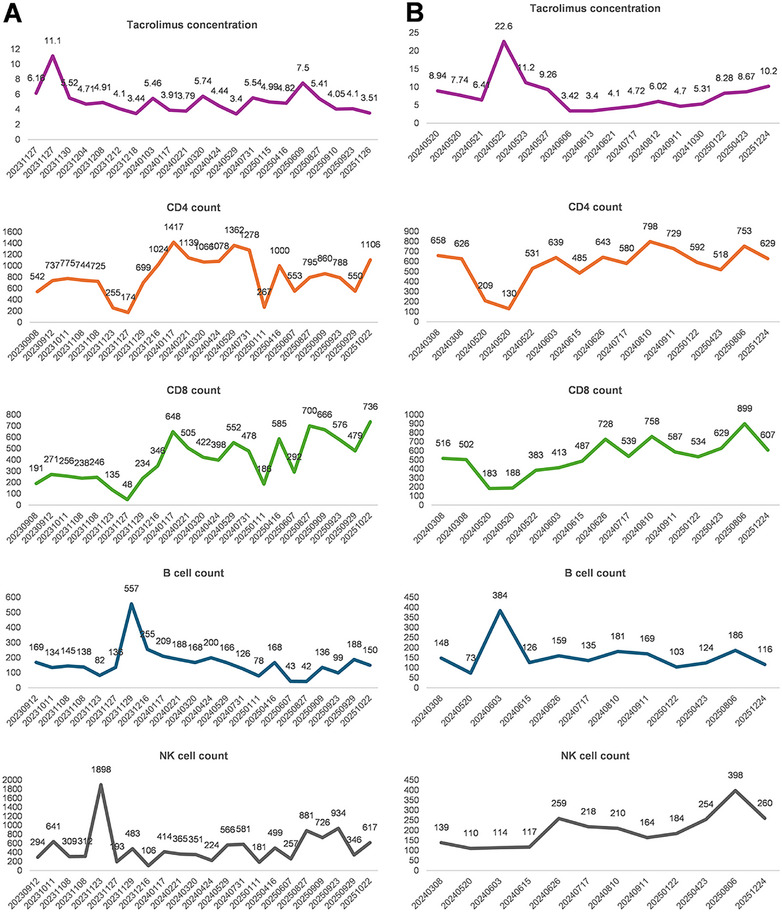
Detailed tacrolimus concentration and serum TBNK in Case 3 (A) and 4 (B).

In contrast, the two recipients with T4 laryngeal SCC (aged 53 and 54 years) who received neoadjuvant chemotherapy combined with immune checkpoint inhibitors achieved favorable recovery without acute or subacute graft rejection, opportunistic infections, or significant metabolic complications in the first 2 postoperative years, with the detailed immunological parameters demonstrated in Figure 1. More importantly, no sign of tumor recurrence or metastasis has been observed till drafting this manuscript.

**FIGURE 2:**
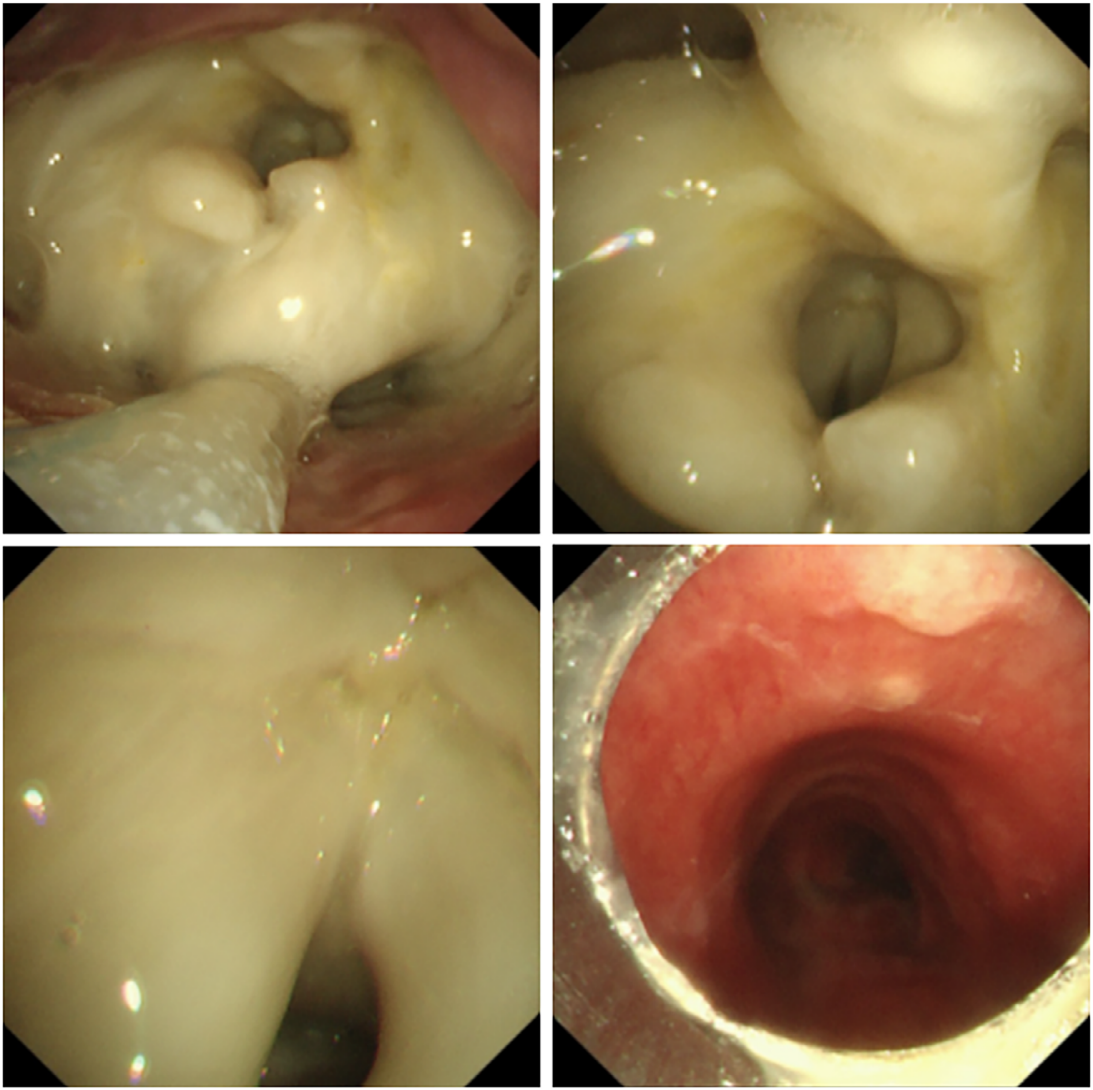
The most recent laryngoscope of Case 3 in December 2025.

However, a rejection episode was noted 3 months prior to drafting this manuscript after Case 3 self‐discontinued methylprednisolone for more than 30 days without notification. The cessation led to signs of rejection, including a pale, malodorous larynx (Figure 2). Immediate reinstitution of methylprednisolone have been initiated to rescue the graft, a nasogastric tube was reinserted to ensure the nutrition, while rigorous monitoring was applied to prevent lethal complications like severe inspirational pneumonia or neck abscess.

### 2.3 Respiratory Function and Airway Management

**FIGURE 3:**
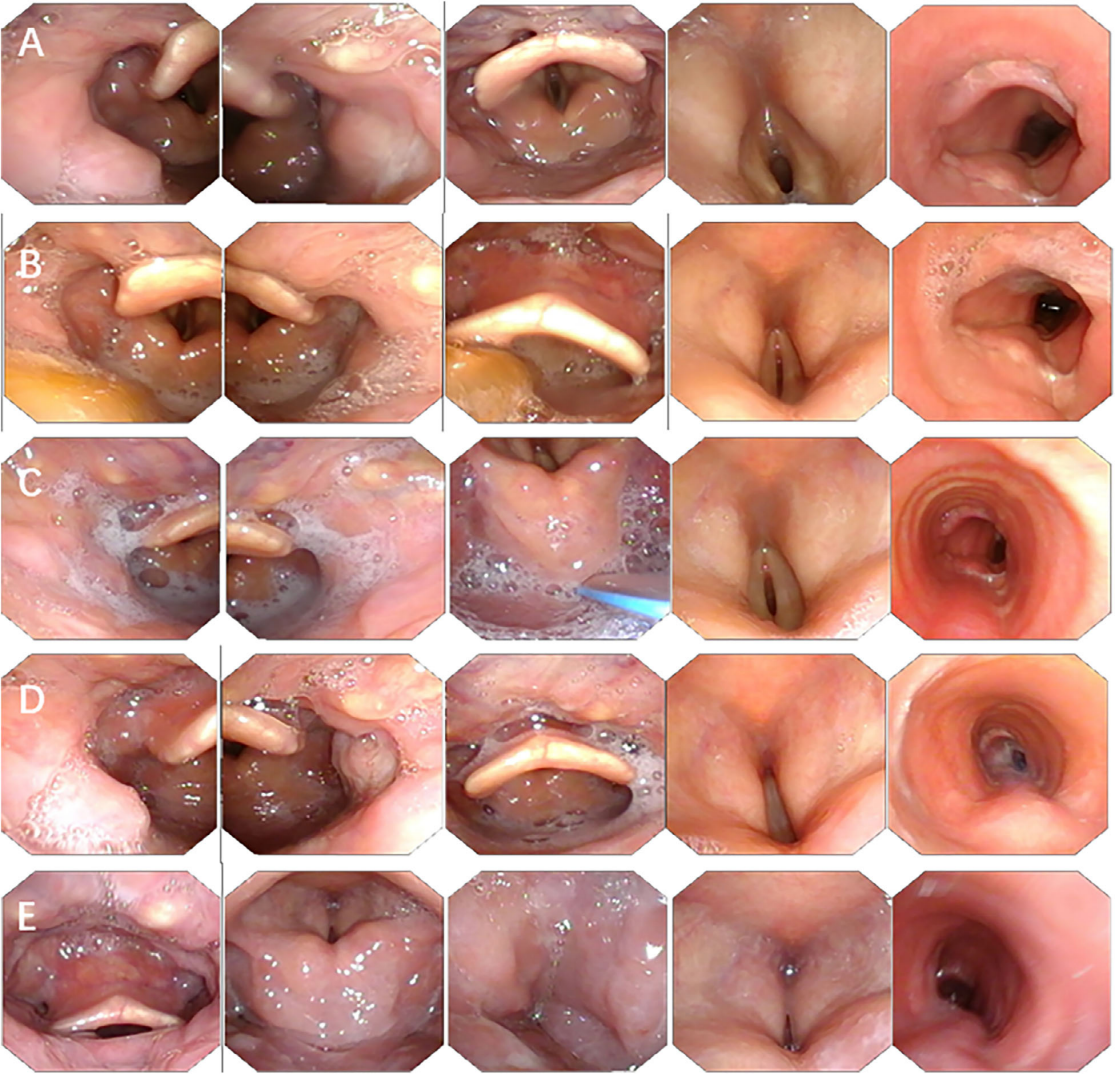
Postoperative laryngoscopy of Case 3. (A) At 3 months postoperatively, significant pyriform sinus residue persisted, necessitating continued NGT‐assisted feeding. (B) By 4 months, pyriform sinus retention showed marked improvement. (C) At 5 months, the NGT was removed with successful transition to oral intake. (D) By 6 months, the vocal cords achieved midline positioning. (E) Further functional progression was observed at 8 months.

Respiratory outcomes faced a specific challenge regarding decannulation, as the vocal cords start to close up starting around Month 3 (Figure 3A). In Cases 3 and 4, recurrent laryngeal nerve (RLN) function restoration was both observed around 4–5 months postsurgery (Figure 3B, C). However, between 6 and 8 months, the vocal cords shifted from paramedian to bilateral medial positions (Figure 3D, E). While this position facilitated phonation, it resulted in respiratory distress upon occlusion of the tracheostomy tube. Consequently, despite the successful restoration of swallowing and speech, decannulation was not achieved by the latest follow‐up (26 and 20 months postsurgery).

### 2.4 Swallowing Function

Swallowing recovery improved progressively across the cohort. The first recipient required laser‐assisted diverticulectomy at 5 months to tolerate a semi‐liquid diet. The second recipient achieved soft diet tolerance by 6 months. The two long‐term survivors achieved complete oral intake by 6 months, allowing for gastric tube removal. Video evidence at 6 months confirmed ideal swallowing function in these patients (Video S1), which remained satisfactory through 23 months for Case 3, who now is suffering from graft rejection and has to return to tube feeding. The good swallowing function has remained for 20 months for Case 4 till the most recent follow‐up visit.

### 2.5 Speech Function

Vocal restoration followed a timeline of reinnervation. Early postoperative phonation was characterized by hoarseness (Video S2). By the 5th to 6th postoperative month, speech clarity improved significantly. The most recent laryngoscope of Case 4 demonstrated good movement of vestibular fold as well as shivering of vocal cords (Video S3). In the two long‐term survivors, the assessed voice quality was encouraging, with fundamental frequency returned to the normal adult male range (123 ± 15 Hz). More recent objective measurements showed a sustained phonation time of 18.49 s of Case 3 and 11.69 s of Case 4, with an *S*/*Z* ratio of 1.22 and 1.62, respectively, indicating enhanced vocal endurance and control.

## 3 Discussions

Given the paucity of available data and protocols [2, 3, 4, 5, 6], our surgical and perioperative experiences with this technique were marked by several challenges. To inform future investigations, we present our findings.

The difficulty in obtaining a high‐quality donor larynx is somehow challenging due to the immature procurement method. Our team's previous research on rabbit and rat larynx transplantation animal models found that if in situ perfusion (before the organ is separated from the donor's body) is used, not only is the amount of perfusion fluid extremely large and the cost is high, but also due to the large number and complex communication of microvessels within the larynx and surrounding tissues, in situ perfusion cannot completely achieve the expected perfusion effect, thereby affecting the transplantation effect. Therefore, we adopted an extracorporeal perfusion for the larynx. We also discover that due to the existence of multiple venous drainage from the muscle and other adjacent tissue, the perfusion can be better achieved using both anterograde and retrograde perfusion. As a result, it is necessary to complete the procurement of the entire multiorgan cluster before perfusing other transplantation organs that require in situ perfusion. Which lead to multiple difficulties: It is necessary to separate and excise the multiorgan cluster in a limited operating space and in the shortest possible time, while distinguishing and protecting the blood vessels and nerves that need to be anastomosed, which is very proficiency demanding. During our four transplantations, after excising the organ with attached internal jugular vein and common carotid artery, perfusion was initiated anterogradely from the artery and subsequently retrogradely from the vein. The entire extracorporeal perfusion process required only a single 1000 mL bag of histidine–tryptophan–ketoglutarate (HTK) solution.

To mitigate the risk of graft vein thrombosis and the potentially fatal consequences of internal jugular vein thrombosis, we departed from previous techniques and implemented a total microvascular transplantation approach. Anatomizing multiple veins ensured adequate circulation, even in the event of venous thrombosis. Although the procedure was slightly prolonged, postoperative Doppler ultrasound examinations revealed optimal blood flow.

In the absence of established graft condition indicators, we rely on the thyroid graft's blood supply as a surrogate marker of graft viability, assessed by Doppler ultrasound. Daily fiber bronchoscopy was conducted to monitor the laryngeal mucosa and ensure effective airway clearance. Regarding graft rejection monitoring, the limited information available in the literature, including Grajek's skin biopsy, is not directly applicable to our case [6]. While serial mucosal biopsies could potentially monitor rejection, they carried the risk of excessive tissue sampling and bleeding complications. To establish a suitable monitoring protocol, we implement a strategy comprising: ① twice‐weekly lymphocyte subpopulation monitoring; ② twice‐weekly multiplex cytokine assays; ③ biweekly laryngoscopy; and ④ Doppler ultrasound examination for blood vessel patency in the immediate postoperative recovery period.

In terms of postoperative management, learning from the negative outcomes of the first two cases, we employ a phased immunosuppression approach combined with a tiered anti‐infective strategy, initiate intraoperatively with intensive immunosuppression to rapidly control T‐cell activation for the latter two cases. Tacrolimus is administered at a loading dose of 1.5–2 mg q12h to achieve short‐term target levels of 10–15 ng/mL, while methylprednisolone is given as a high‐dose intravenous infusion (200 mg/day) and tapered stepwise to 20 mg/day over 7 days to suppress acute inflammatory responses. To further block IL‐2‐mediated immune activation, basiliximab (20 mg IV) is administered both intraoperatively and on postoperative Day 4, creating a triple induction barrier. During the maintenance phase, immunosuppression is dynamically optimized through close monitoring, with tacrolimus levels strictly maintained at 5–8 ng/mL to balance efficacy and renal toxicity risks. Mycophenolate mofetil (MMF) dosing is adjusted between 0.5 and 0.75 g q12h to achieve a target MPA (mycophenolic acid) AUC_0–12h_ (Area under curve) of 35 mg h L^−^^1^, ensuring adequate immunosuppression, while methylprednisolone is gradually transitioned to a low oral maintenance dose (5 mg/day) to minimize long‐term side effects. This phase emphasizes personalized dosing through therapeutic drug monitoring to maintain immune homeostasis. However, due to suboptimal compliance of drug use of Case 3, the graft suffered from severe acute rejection, which may eventually result in graft loss. Our plan for graft loss was to conduct a TL along with jejunal flap reconstruction of the pharynx and esophagus; however, the vascular anastomosis can be tricky due to the complex surgical history of the neck.

For infection control, a multilayered prevention and treatment strategy is implemented for the latter two cases, strictly differentiating between prophylactic and therapeutic interventions. Prophylaxis centers on broad‐spectrum coverage with piperacillin–tazobactam for Gram‐negative and anaerobic bacteria, combined with Trimethoprim/sulfamethoxazole (TMP–SMX) for Pneumocystis pneumonia (PCP) prevention, while avoiding routine antiviral or antifungal prophylaxis. In cases of suspected infection (fever >38.5°C or elevated biomarkers), targeted therapy is initiated only after pathogen identification through blood cultures, sputum analysis, or metagenomic next‐generation sequencing (mNGS). Drug interactions are carefully managed, including monitoring tacrolimus–azole synergism, renal dose adjustments for ganciclovir, and avoiding concomitant use of sulfonamides with MMF to mitigate myelosuppression risks. This “prevention–diagnosis–targeted therapy” approach minimizes unnecessary antimicrobial exposure, reducing resistance development and drug toxicity.

Pharmacodynamic monitoring is integral to clinical decision‐making, with weekly tacrolimus level monitor during the first postoperative month (transitioning to monthly once stable) and monthly MMF pharmacokinetic profiling via limited sampling (0, 2, 4, and 6‐h postdose measurements). Infection biomarkers including procalcitonin (PCT), C‐reactive protein (CRP), and β‐d‐glucan are tracked serially to guide real‐time adjustments to anti‐infective regimens. This comprehensive monitoring system integrates immunosuppression intensity, infection risk, and organ function parameters to maximize therapeutic precision and safety. The protocol's effectiveness is demonstrated in the third and fourth recipient, who remained free of rejection, opportunistic infections, or tumor recurrence through 23 and 20 months of follow‐up, validating both the safety and efficacy of this management strategy.

The functional success of laryngeal transplantation is contingent upon the fidelity of reinnervation, particularly of the recurrent laryngeal nerve (RLN). Unlike solid organ transplants where autonomic reinnervation is incidental, the larynx requires precise somatic motor recovery for the conflicting tasks of phonation (adduction) and respiration (abduction) [15]. In terms of reinnervation, the RLN's mixed composition makes it uniquely susceptible to misdirected regeneration compared with purely motor nerves. In our study, all patients remained cannulated due to insufficient reinnervation of RLN, which is common in clinical practice, while the majority of patients can only regain part of the nerve function, while the vocal cords are generally fixed in median position [16]. Future surgery could be modified to avoid bilateral incomplete paralysis of vocal cord, which leads to tracheostoma‐dependent, unilateral RLN anastomosis could be attempted in order to achieve decannulation at the sacrifice of some speech function.

While short‐term graft survival is achievable, the long‐term risk profile of laryngeal transplantation in cancer patients is dominated by the complex interplay between immunosuppression and tumor surveillance. The primary concern is the potential for calcineurin inhibitor‐mediated tumorigenesis [9]. Agents such as tacrolimus have been shown to upregulate transforming growth factor‐β (TGF‐β) and vascular endothelial growth factor (VEGF) expression, potentially creating a pro‐oncogenic microenvironment that accelerates the growth of occult micrometastases [10]. Epidemiological data from solid organ transplantation confirm a significantly elevated risk of de novo SCCs in immunosuppressed cohorts [17]. Consequently, we advocate for a minimization strategy for maintenance immunosuppression, just like the goal set for tacrolimus concentration at 5–8 ng/mL to balance graft viability with oncologic safety.

Our study is subject to several limitations inherent to the novelty and complexity of laryngeal transplantation. The sample size (*n* = 4) is small; consequently, the study is descriptive in nature, while the limited cohort size precludes meaningful inferential statistical analysis, and no attempts were made to calculate statistical significance or predictive power. Therefore, outcomes regarding graft survival and functional recovery should be interpreted as preliminary proof‐of‐concept data rather than generalized clinical evidence. Future multicenter studies with larger cohorts will be essential to establish statistical validity and to refine the prognostic factors for patient selection.

In conclusion, the complexity of laryngeal transplantation, particularly in cancer patients, necessitates further investigation of surgical techniques, perioperative management, and long‐term outcomes. Our study demonstrates the application of laryngeal transplantation in four cancer patients, with microvascular anastomosis and extracorporeal perfusion proving safe and effective. We also provide an adaptable approach that provides a replicable model for laryngeal transplantation, emphasizing dynamic monitoring and individualized therapy to optimize outcomes. However, further validation through multicenter studies with extended follow‐up will help refine its broader applicability; longer follow‐up period might be essential for monitoring the tumor recurrence.

## 4 Methods and Materials

This study was conducted between 2023 and 2024 at West China Hospital. A prospective case series of four patients with either laryngeal or hypopharyngeal cancers that had LT and subsequent larynx–hypopharynx–esophagus–thyroid–parathyroid–trachea transplantation was performed. All patients gave informed consent. Data regarding tumor pathology, prior treatments, staging, recurrence, intraoperative and postoperative complications, and long‐term function outcomes were collected. This study was approved by the West China Hospital's institutional review board with approval number of (No. 2017[362]). The clinical trial was registered with the registration number of ChiCTR2400080222.

### 4.1 Inclusion and Exclusion Criteria

Inclusion criteria: (1) Pathologically confirmed laryngeal cancer or hypopharyngeal cancer or tongue cancer without distant metastasis requiring LT should meet the following criteria: (a) patients who have already undergone LT for laryngeal cancer or other diseases; (b) patients who are under 75 years of age and have urgent requirements for vocal function restoration; (c) patients with well baseline conditions; (d) patients are willing to take lifelong immunosuppressive drugs; (2) patients with severe laryngotracheal stenosis; patients must meet the following criteria: (a) patients with severe laryngotracheal stenosis who are unable to undergo decannulation after at least one failed surgical attempt; (b) patients who are less than 75 years of age and have urgent requirements for vocal function; (c) patients with good general baseline conditions; (d) patients who are willing to take lifelong immunosuppressive drugs; (3) locally advanced thyroid cancer; enrolled patients must fulfil the following criteria: patients with recurrence after at least one surgery requiring total laryngeal and long‐segment tracheal (esophageal) resection.

Exclusion criteria: (1) patients with advanced laryngeal cancer, hypopharyngeal cancer, and tongue cancer cannot be enrolled if they have: (a) distant metastasis; (b) older than 75 years of age and do not have urgent requirements for vocal function; (c) poor general condition who cannot undergo laryngotracheal transplantation and lifelong immunosuppressive therapy; (2) patients with severe laryngotracheal stenosis will not be enrolled if they: (a) are older than 75 years of age and have no urgent need for vocal function; (b) have poor general condition and cannot afford laryngotracheal transplantation and lifelong immunosuppressive therapy; (c) patients who can have their narrow laryngotracheal lumen restored by conventional surgery;

### 4.2 Equipment

The solution used for organ perfusion and store was custodiol HTK solution (Köhler Chemie GmbH, Germany). The sutures used for skin or tissue were fast‐absorbable multifilament polyglactine suture (Vicryl 4/0 or 3/0, Ethicon Inc; Johnson & Johnson, Somerville, NJ, USA). The sutures used for vascular and nerve anastomosis were nonabsorbable monofilament polypropylene suture (Prolene 8/0, Ethicon Inc; Johnson & Johnson). The microvascular anastomotic coupler for venous anastomosis was GEM Microvascular Anastomotic Coupler Device (Synovis Micro Companies Alliance, Birmingham, AL, USA).

### 4.3 Patient Selection and Preoperative Assessment

Patient eligibility was determined through a rigorous multidisciplinary review involving head and neck surgeons, transplant specialists, oncologists, and ethicists, while strictly adhering to the registered protocol (ChiCTR2400080222). The study protocol, including the risks of immunosuppression‐mediated cancer progression, was strictly screened and approved by the institutional review board. Written informed consent was obtained from all patients and their families after extensive counseling regarding the experimental nature of the procedure and the potential trade‐off between functional quality of life and oncologic surveillance.

Patients were matched for ABO blood type compatibility, human leukocyte antigen, and serum crossmatch negative. After successful donor matching, the patient underwent a comprehensive series of preoperative examinations including laryngoscopy, contrast CT scans of the neck and chest, ultrasounds of the thyroid, heart, abdomen, and urological system and preoperative blood tests. Strict oncologic exclusion criteria were applied to minimize recurrence risk: candidates underwent high‐resolution whole‐body positron emission tomography–computed tomography (PET–CT) to rule out distant metastasis and confirm resectability. After excluding metastasis and other surgical contraindications, the patient underwent laryngeal transplantation, involving the transplantation of the larynx, hypopharynx, thyroid, parathyroid glands, cervical trachea, and esophagus.

### 4.4 Organ Donor Selection

Donors were identified and screened in strict accordance with the institutional protocols of the Red Cross of China and the Ethics Committee of West China Hospital. Eligible donors were brain‐dead individuals, matched to recipients based on ABO blood group compatibility and anthropometric parameters to ensure appropriate laryngeal caliber and graft fit. Rigorous exclusion criteria were applied, including a history of head and neck malignancy, previous laryngeal trauma, or active systemic infection. To ensure the functional integrity of the allograft, all potential donors underwent bedside fiberoptic laryngoscopy to confirm normal laryngeal anatomy and rule out intubation‐related injury. Final procurement proceeded only after obtaining informed consent from the donor's legal next of kin and confirming a negative lymphocytotoxic crossmatch.

### 4.5 Surgical Techniques (In General)

**FIGURE 4:**
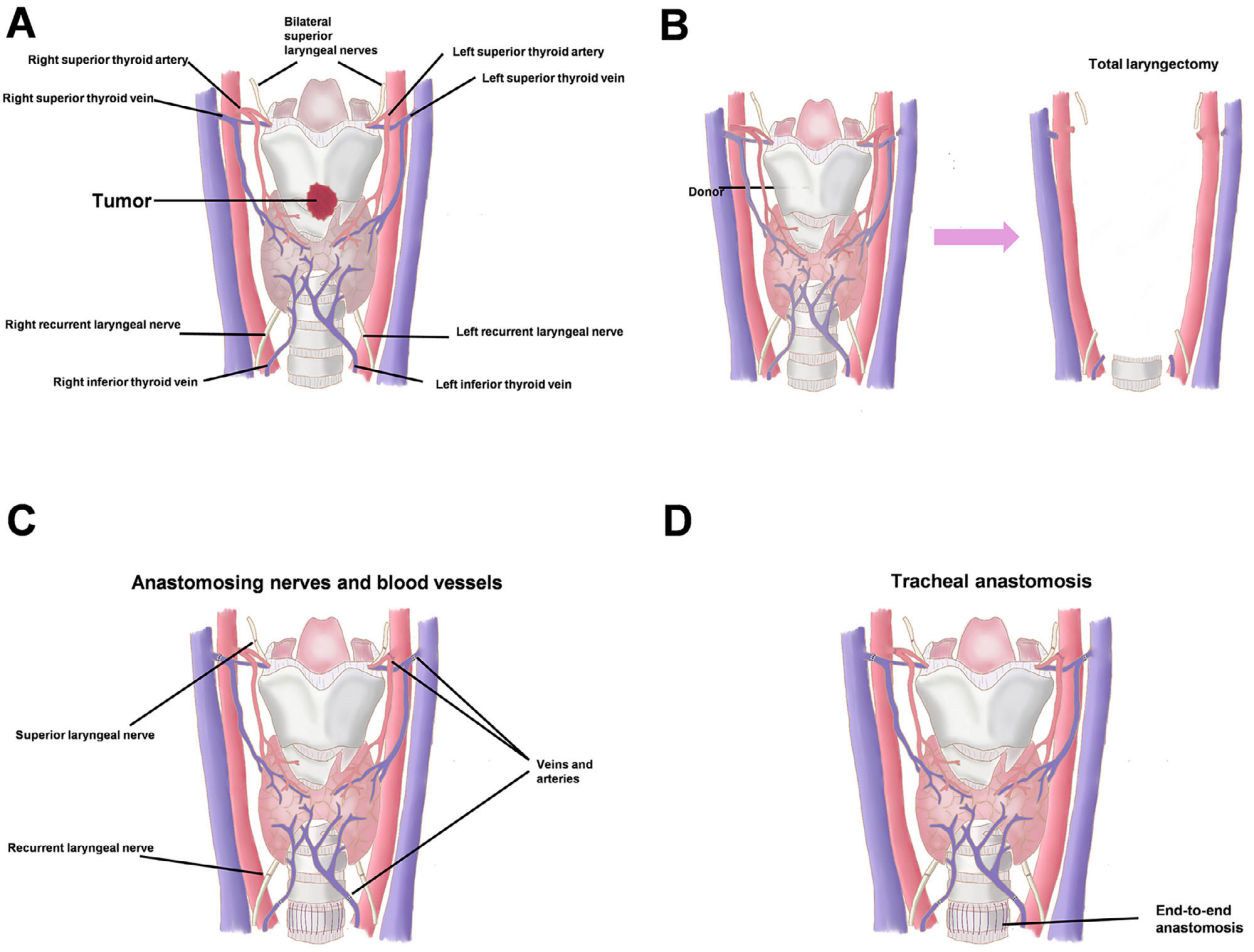
Schematic illustration of the surgery. (A) The anatomical illustration of larynx. (B) Narrow field laryngectomy and larynx transfer. (C) Blood vessel and nerve anastomosis. (D) Tracheal anastomosis.

**FIGURE 5:**
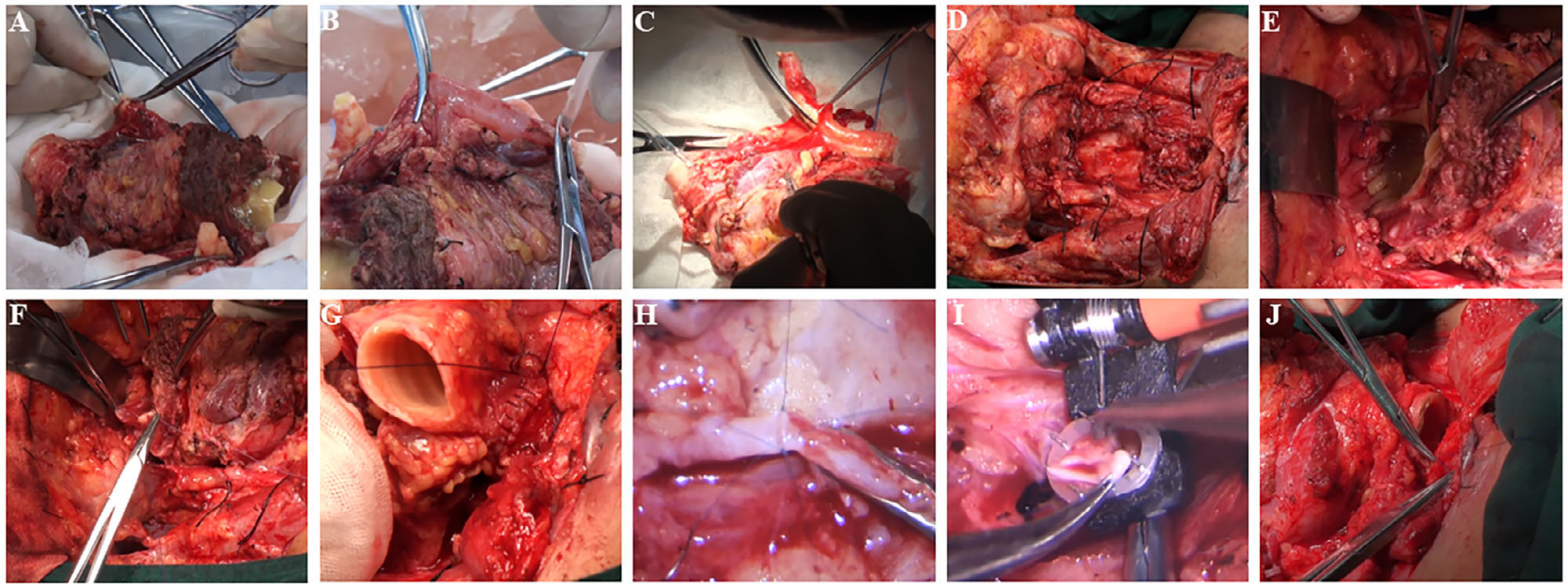
Detailed surgical procedure of Case 3. (A) The larynx was extracorporeally perfused using HTK solution anterogradely through carotid artery. (B) The larynx was extracorporeally perfused using HTK solution retrogradely through jugular vein. (C) Trimming the larynx, excising the major arteries and veins. (D) Narrow‐field laryngectomy with vascular and nerve protection. (E) Anchoring the larynx to the recipient's hypopharynx posteriorly. (F) Anchoring the thyroid cartilage to the recipient's tongue root anteriorly. (G) Esophageal anastomosis. (H) Arterial anastomosis. (I) Venous anastomosis. (J) Tracheal anastomosis.

The whole surgical procedure were detailed in Figures 4 and 5.

The organ procurement was conducted simultaneously with the narrow‐field laryngectomy. The entire larynx, hypopharynx, esophagus, thyroid gland, parathyroid glands, and at least four tracheal rings were procured (Figure 5A). Common carotid artery, internal jugular vein and the attached bilateral superior thyroid arteries, superior thyroid veins were anatomized and protected. The bilateral RLNs and superior laryngeal nerves were also preserved for subsequent anastomosis. After the declaration of clinical death, the complete larynx was removed from the donor and extracorporeally perfused using HTK solution anterogradely through common carotid artery and then retrogradely through internal jugular vein (Figure 5B). After sufficient perfusion, the organ was stored in an ice‐solution mixture and transported to the recipient's operating room. The organ was then trimmed with the bilateral common carotid arteries and right internal jugular vein excised and all the essential microvessels and nerves were labeled to facilitate the anastomosis, the left internal jugular vein was kept due to the small caliber of the left superior and inferior thyroid vein (Figure 5C).

The recipient underwent a narrow‐field laryngectomy with a bilateral elective neck dissection; LT with en bloc resection of the hyoid bone, cricoid cartilage, hypopharynx, first and second tracheal rings, bilateral thyroid and parathyroid glands, and the esophageal inlet was conducted, with preservation of the bilateral superior and inferior thyroid arteries and veins, facial arteries and veins, lingual artery pedicles, and the bilateral superior laryngeal nerves and RLNs (Figure 5D). Frozen section analysis of surgical margins was performed intraoperatively to ensure R0 resection prior to graft implantation.

The larynx was primarily secured by suturing the donor and recipient hypopharynx. The donor's thyroid cartilage was fixed to the recipient's tongue root (Figure 5E, F), and the donor's esophagus was anastomosed to the recipient's esophagus (Figure 5G). The above‐mentioned anastomosis was conducted using 3/0 Vicryl. The blood vessel and nerve anastomosis started from right side to left side and from top to bottom, the donor's right superior laryngeal nerve was anastomosed to the recipient's superior laryngeal nerve using 8/0 prolene. The donor's right superior thyroid artery and vein were anastomosed to the recipient's right superior thyroid artery and vein, arterial anastomosis was conducted using 8/0 prolene, while venous anastomosis used 4.0 mm GEM coupler. And finally, the right RLN was anastomosed using 8/0 prolene. Before starting the anastomosis on the left side, albumin solution was injected into the left superior thyroid artery to flush out the remaining HTK solution from the larynx. The left side anastomosis started with left superior laryngeal nerve using 8/0 prolene, then anastomosing the donor and recipient's superior thyroid artery using 8/0 prolene. After completing the left side arterial anastomoses, the arteries were unclipped to let blood flush the whole vasculature. Then the donor's veins was anastomosed to the recipient's corresponding veins in an end‐to‐side pattern using GEM coupler. And finally, the donor and recipient's RLNs were anastomosed together using 8/0 prolene (Figure 5H, I). Following completion of blood vessel and nerve anastomoses, the donor's trachea was sutured to the recipient's third tracheal ring using 3/0 Vicryl (Figure 5J), while preserving the previous tracheostomy. Thorough hemostasis was achieved, and the surgical procedure concluded by closing the subcutaneous tissue using 3/0 Vicryl and skin using 4/0 Vicryl.

### 4.6 Postoperative Management (In General)

Postoperatively, the patient received standard triple immunosuppressive therapy consisting of methylprednisolone sodium succinate, tacrolimus, and MMF. Doses were dynamically adjusted according to the serum tacrolimus trough level at around 5–8 ng/mL, MMF metabolite concentration at around MPA AUC_0–12h_ of 35 mg h L^−^^1^, and immune function parameters including CD3+, CD4+, and CD8+ T‐cell subsets as well as cytokines such as interleukin‐6 (IL‐6) and tumor necrosis factor‐α (TNF‐α). A combined anti‐infection regimen was implemented for comprehensive prophylaxis covering bacterial, viral, and fungal pathogens: piperacillin–tazobactam for antibacterial prophylaxis, ganciclovir for cytomegalovirus prevention, trimethoprim–sulfamethoxazole for PCP prophylaxis, and caspofungin for antifungal coverage.

The postoperative rehabilitation program was individually tailored and dynamically adjusted based on real‐time vocal function assessments, incorporating neck muscle exercises, postoperative voice protection education, and targeted neuromuscular electrical stimulation combined with laryngeal motor coordination training. Systematic voice therapy was implemented concurrently to facilitate vocal function recovery, complemented by psychological interventions to enhance social adaptation. Family members and friends were actively encouraged to participate in the rehabilitation process. This comprehensive program integrated laryngeal functional reconstruction with neuromuscular adaptation, specifically including: (1) laryngeal relaxation training using cricothyroid muscle massage coordinated with head–neck movements to reduce compensatory laryngeal muscle tension during phonation, with massage intensity progressively adjusted according to neck wound healing; (2) respiratory–phonatory coordination training employing intermittent tracheostomy stoma occlusion to induce diaphragmatic breathing patterns, where therapists guided patients to perceive abdominal muscle contraction during phonation, while family members supervised progressive exercises from monosyllables to paragraph reading; (3) resonance enhancement training initiating with nasal /m/ sounds to guide patients in perceiving lip–palate–facial vibration transmission, establishing low laryngeal pressure phonation to minimize vocal fold trauma. Based on recovery outcomes from the first two cases, the research team continuously refined training strategies for subsequent cases, particularly focusing on strengthening vocal fold musculature and promoting RLN reinnervation.

### 4.7 Outcome Assessment

The primary outcomes of this study were rejection‐free survival, which is defined as the length of time after an organ or tissue transplant during which the recipient does not experience any episodes of graft rejection. And disease‐free survival, which is defined as the length of time after surgery, that a patient survives without any detectable signs or symptoms of cancer, was evaluated on a routine basis after surgery (once a month in the first 6 months, then every 3 months). Blood test for tacrolimus concentration and immunological profile was collected on each visit, laryngoscopy for potential graft rejection was done every 3 months, while CT scan and laryngoscopy for potential locoregional recurrence was done every 3 months.

Secondary outcomes included voice, speech, and respiration function evaluations. The voice, swallowing, and respiration functions of laryngeal transplant patients was evaluated at 1, 3, 6, and 8 months and then every 3 months after surgery. The voice and swallowing were assessed in an objective manner, while the respiration was assessed mainly in subjective manner, mainly relied on the patient's reported feeling with the assistance of the observed glottal width under laryngoscopy.

The objective evaluation of voice function included acoustic analysis and laryngoscopic analysis. Acoustic signals were recorded with a sampling rate at 44.1 kHz and analyzed with the lingWAVES software (German, version 2.0). Five objective parameters of voice quality were measured: fundamental frequency (F0), maximum phonation time (MPT), jitter (index of F0 variability), shimmer (index of intensity variability), and the harmonics to noise ratio (HNR). OLYMPUS Enf‐p4 rhinolaryngoscope (Olympus America Inc., Westborough, MA, USA) was employed to evaluate the morphology and mobility of the vocal cords.

The objective evaluation of swallowing function was conducted under fiberoptic endoscopic evaluation of swallowing examination; dysphagia severity assessment was conducted according to the Yale Pharyngeal Residue Severity Rating Scale (YPRSRS) [18].

## Author Contributions

Conceptualization: F.C. and J.L. Methodology: Z.J., F.C., J.L., L.W., M.A., and X.Y. Investigation: Z.J., M.A., J.L., Z.B., X.Y., Y.H., L.W., P.Z., and F.C. Visualization: Z.J., M.A., P.Z., Y.H., and X.Y. Funding acquisition: F.C. and J.L. Project administration: Z.J., F.C., and J.L. Supervision: F.C., J.L., L.W., and Y.X. Writing – original draft: Z.J., M.A., and P.Z. Writing – review and editing: Z.J., M.A., Y.X., X.Y., Y.H., P.Z., F.C., L.W., and J.L. All authors have read and approved the final manuscript.

## Funding

This research has been funded by the 1.3.5 Project for Disciplines of Excellence, West China Hospital, Sichuan University (grant number: 25HXJS001) and Chinese Academy of Medical Sciences (CAMS) Seed Program for Clinical and Translational Medicine (grant number: 2024‐I2M‐C&T‐B‐095)).

## Conflicts of Interest

The authors declare no conflicts of interest.

## Ethics Statement

The study has been approved by the official institutional review board of West China Hospital with approval number of No. 2017[362]. Registered with Chinese Clinical Trial Register with a number of ChiCTR2400080222.

## Supporting information




**Video S1**. The swallowing function of case 3 at 6‐month follow‐up.

**Video S2. The** speech function of case 3 at 6‐month follow‐up.

**Video S3**. **The** laryngoscope of case 4 at 19 month follow‐up.‐

**Supporting File 1**: mco270748‐sup‐0001‐SuppMat.docx

## Data Availability

The majority of the data are presented in the article, more detailed data can be obtained by emailing the corresponding author.
