# Supplementary material for: Laryngeal Transplantation in Cancer Patients: Evaluation of Surgical Outcomes and Functional Recovery
Source: MedComm (2020). 2026 Apr 26;7(5):e70748. doi: 10.1002/mco2.70748 (PMC13111920; doi:10.1002/mco2.70748)
Supplement: Supplementary file 4 — Supporting File 1: mco270748‐sup‐0001‐SuppMat.docx [file MCO2-7-e70748-s003.docx]

**Laryngeal Transplantation in Cancer Patients: Evaluation of Surgical Outcomes and Functional Recovery**

Zheng Jiang^1, 2, 3, 4#^, Mailudan Ainiwaer^1, 2#^, Pengwei Zhao^1, 2#^, Yansheng Hu^1, 2^, Xin Yang^1, 2^, Yu Xiong ^1, 2^, Bin Zeng^1, 2^, Longhao Wang^1, 2*^, Jun Liu^1, 2*^, Fei Chen^1, 2*^

^1^ Department of Otolaryngology-Head & Neck surgery, West China Hospital, Sichuan University, Chengdu, China.

^2^ Head and Neck Surgical Center, West China Hospital, Sichuan University, Chengdu, China.

^3^ Department of Biotherapy, West China Hospital, Sichuan University, Chengdu, China.

^4^ West China Lecheng Hospital, Sichuan University, Qionghai, China.

* Equal contributors.

**Correspondence:**

**Longhao Wang**: Email: entwlh@outlook.com; Affiliation: West China Hospital, Sichuan University; Address: 37 Guoxue Lane, Chengdu, Sichuan Province, PRC.

**Jun Liu:** Email: hxheadneckjunl@163.com; Affiliation: West China Hospital, Sichuan University; Address: 37 Guoxue Lane, Chengdu, Sichuan Province, PRC.

**Fei Chen**: Email: hxchenfei@163.com; Affiliation: West China Hospital, Sichuan University; Address: 37 Guoxue Lane, Chengdu, Sichuan Province, PRC.

**Supplementary Videos**

**Video S1.** The swallowing function of case 3 at 6-month follow-up.

**Video S2.** The speech function of case 3 at 6-month follow-up.

**Video S3**. The laryngoscope of case 4 at 19 month follow-up.-
